# One step closer to a universal influenza A vaccine?

**Published:** 2011-01-10

**Authors:** Leo LM Poon

**Affiliations:** 1 Department of Microbiology, The University of Hong Kong, Hong Kong SAR

## 

The continuous antigenic drifts and occasional antigenic shifts enable human influenza viruses to escape the human immune system. Moreover, the frequent occurrence of human H5N1-infected cases and the recent emergency of a novel swine-like human H1N1 influenza virus further reiterate the risk of the introduction of a new pandemic strain to humans through *in toto* transfer of animal influenza viruses. The discovery of neutralizing antibodies that are broadly reactive with multiple influenza subtypes is therefore extremely important for the influenza pandemic preparedness, for use either for therapeutic purposes or as the basis of vaccine development. On the other hand, recently studies also suggest that cell-mediated immunity might be critical for cross-subtype protection against influenza virus infection. Using vaccinia virus-based H5 vaccine, we also demonstrated this approach might also capable of inducing cross-subtype protection. Mice received this H5 vaccine had no detectable neutralization antibody against other HA subtypes, suggesting that cell-mediated immunity might be account for the subtype protection. Here, we would discuss our recent findings on these research topics.

